# Antileukemic potential of Nile blue-mediated photodynamic therapy on HL60 human myeloid leukemia cells

**DOI:** 10.55730/1300-0152.2662

**Published:** 2023-06-17

**Authors:** Serçin ÖZLEM ÇALIŞKAN, Aynur KARADAĞ GÜREL, Barış UZUNOK, Numan TAŞPINAR, Berna AKIN, Metin ÇALIŞKAN, Rahşan ILIKÇI SAĞKAN

**Affiliations:** 1 Department of Biophysics, Faculty of Medicine, Uşak University, Uşak, Turkiye; 2 Department of Medical Biology, Faculty of Medicine, Uşak University, Uşak, Turkiye; 3 Department of Physiology, Faculty of Medicine, Uşak University, Uşak, Turkiye; 4 Department of Pharmacology, Faculty of Medicine, Uşak University, Uşak, Turkiye; 5 Department of Molecular Biology and Genetic, Faculty of Science, Uşak University, Turkiye

**Keywords:** Nile blue, photodynamic therapy, leukemia, apoptosis

## Abstract

**Background/aim:**

Photodynamic therapy (PDT) has received great attention over the past decade in the treatment of diseases such as leukemia which is a cancer of the blood and bone marrow cells that causes a significant number of deaths worldwide. In this study, it was aimed to investigate the effects of Nile blue-mediated PDT (NB-mediated PDT) on HL60 cells.

**Materials and methods:**

The effect of NB-mediated PDT on cell proliferation was evaluated with cell volume analysis using flow cytometry at 24 h. Cell apoptosis, ROS production, mitochondrial membrane potential, and cell cycle analysis were evaluated using annexin V-FITC, H_2_DCFDA, JC-1, and PI staining, respectively, by flow cytometry and fluorescence microscopy. The morphological and ultrastructural analyses were examined by Giemsa staining and SEM. CD11b staining is used to determine the differentiation of leukemia cells.

**Results:**

NB-mediated PDT induced an apoptotic response at 12.5 μM in HL60 cells. When Giemsa staining and SEM images were evaluated, apoptotic bodies, holes, and occasional folds were detected on the surfaces of cells in the NB-mediated PDT group.

**Conclusion:**

The NB-mediated PDT had no effect on the differentiation of leukemia cells, but this therapy affects the growth of HL60 cells in vitro, which may provide a new idea for removing leukemic cells from bone marrow intended for autologous transplant.

## 1.Introduction

Photodynamic therapy (PDT) has received great attentionover the past decade for cancer therapy applications suchas leukemia, which is a cancer of the blood and bonemarrow cells that causes a significant number of deathsworldwide. This problem has led to an increasing interestin the discovery of novel treatment options that will beeffective against leukemia. Photodynamic therapy is anoninvasive therapeutic method based on the simultaneous combination of suitable light and a photosensitizer,and the generation of reactive oxygen species in cellsin the presence of molecular oxygen (Hamblin, 2020).Photodynamic therapy requires the combination ofthree different components: photosensitizer, light, andmolecular oxygen. Individually, these components arenot toxic; when combined, they form cytotoxic reactiveoxygen species. PDT has been approved for use in manycountries for various types of cancer and actinic keratosis, Barrett’s esophagus, atherosclerotic vascular disease, age-related macular degeneration, etc. (Fayter et al., 2010). Compared to traditional therapeutic methods, PDT offers major selectivity against tumor cells due to the use of photosensitizers localized in tumor lesions (Correia et al., 2021). Porphyrin and nonporphyrin photosensitizers have been included in clinical studies; and in fact, many of them have been approved for application in oncology. Photofrin, Purlytin, Foscan, Lutrin, NPe6, Levulan, Tookad, Metvix, ADPM06, Hypericin, Cyanines Methylene blue, Toluidine blue, and Nile blue derivatives are among the photosensitizers (O’Connor et al., 2009). Nile blue is a benzophenoxazine dye with a maximum absorption of light at 600–650 nm range, is effective in PDT, and shows promising applications for cancer treatment and tumor diagnosis owing to its low systemic toxicity. NB is a cationic dye by nature and exhibits a high binding affinity for DNA, which is an important target in cancer cells (Lin et al., 1991a; Lin et al., 1991b; Lin et al., 1993; Tong et al., 2001). Moreover, its phototherapeutic effect on leukemia and the underlying mechanisms of action, such as cell differentiation remains unclear. This circumstance encouraged the investigation of the therapeutic possibility of using NB-mediated PDT on human acute myeloid leukemia (AML) HL60 cells.

In our ongoing efforts to identify and understand the modes of action of novel and effective antileukemic agents, several photosensitizers are currently being evaluated in our laboratory. Autologous bone marrow transplantation is one of the methods used in the treatment of leukemia, but tumor cell contamination in autografts affects transplantation success. Nile blue analogues preferentially localize to lysosomes and are also DNA sensitizers. The effects of leukemia cells underlying this enhanced PDT-mediated cell killing by the lysosome-directed photosensitizers are not clear. The photosensitizing effect of photosensitizers used in photodynamic therapy by penetrating into malignant cells may be a helpful method in purification of bone marrow. Here, the mechanistic aspects of NB-mediated PDT induced cell death and cell differentiation were reported in the HL60 cell line. The physiological and ultrastructural alterations were also investigated in the HL60 cell line. This study reveal that it is capable of inducing leukemia cell death by mitochondrial dysfunction, reactive oxygen species generation, apoptotic features such as phosphatidylserine externalization, subG1 peak detection, apoptotic cell morphology, and ultrastructure. We also found that NB-mediated PDT had no effect on HL60 cell differentiation by CD11b staining. Nile blue, which is a lysosome-targeted photosensitizer and DNA sensitizer, was enhanced by PDT-mediated cell killing for leukemia cells in vitro.

## 2. Materials and methods

### 2.1. Cell culture

HL60 myeloid leukemia cell line was cultured in RPMI-1640 medium, supplemented with 10% fetal bovine serum (FBS), incubated at 37 °C in a CO_2_ incubator.

### 2.2. Photosensitizer

NB was used as a photosensitizer in this study. A stock solution of NB was prepared in ethanol. Various concentrations of NB (200, 100, 50, 25, 12.5, 6.25, 3.25, and 1.56 μM) were used for NB-mediated PDT to determine the final experimental concentration. At the end, final experimental NB concentrations were specified as 1.56, 3.25, 6.25, 12.5, 25 μM because of observing any living cells over 50 μM concentration. Stock solutions of NB were diluted in RPMI medium containing 10% FBS. The final volume was adjusted to 1 mL with RPMI medium for each well of a 24-well microplate. A total of 3 × 10^5^ cells were exposed to NB in the dark for 1 hour at 37 °C.

### 2.3. Study design

The effects of NB-mediated PDT on HL 60 cells at different concentrations were studied in vitro. Experimental groups were formed as follows:

1. Control group: No NB, no light,
2. Only light group: only treated with light,
3. Only NB group: NB in all concentrations that were not irradiated,
4. NB-mediated PDT group: irradiated in the presence of NB.

### 2.4. PDT application

After the cells were exposed to the Nile blue for 1 h, the cells were centrifuged at 1000 rpm for 5 min, and the medium containing free Nile blue was removed. The cells were washed three times with PBS, and fresh PBS was added onto the samples. The cells were then transferred to a 24-well plate and then exposed to a light source. The light source was an LED (O’melon Omega Led) system containing 283 units (in a three-panel) that emit red light, with 640 nm wavelength. The light output was measured by a power meter (Newport, USA) and delivered an irradiance of 0.47 mW/cm^2^ and a fluence of 0.84 J/cm^2^ at 30 min.

### 2.5. Cell viability and cell volume analysis

For proliferation assay, cell viability was quantified by the trypan blue exclusion method. Cells were plated in 96-well plates. At the end of treatment, 50 μL of 0.4% trypan blue dye was added to 50 μL of cell suspension and mixed by pipetting. After 1–2 min of incubation at room temperature, the blue cells and viable cells were counted on a hemocytometer. The viable cell count was calculated as % viable cells = [1.00 – (number of blue cells ÷ number of total cells)] * 100. A cell volume assay was used to confirm the cytotoxic effect of NB-mediated PDT. A rapid and sensitive method was established for the measurement of cytotoxicity using the flow cytometry analysis system. The study developed by Winkelmeier et al. to determine the cytotoxic effect based on cell volume measurement was adapted to the flow cytometric system (Winkelmeier et al., 1993). Various concentrations of NB (25, 12.5, 6.25, 3.25, and 1.56 μM) were used for NB-mediated PDT to determine final experimental concentration. At the end, final experimental NB concentrations were specified as 1.56, 3.25, 6.25, 12.5, 25 μM because concentrations of over 50 μM had a toxic effect. Stock solution of NB was diluted in RPMI medium containing 10% FBS. The final volume was adjusted to 1 mL with RPMI medium for each well of a 24-well microplate. Next, 3 × 10^5^ cells were exposed to NB in the dark for 1 h at 37 °C. Histogram and dotplot graphics were performed, FSC-H which represents the cell volume. A total of 10,000 events were acquired in the region that corresponded to the HL60 leukemia cells.

### 2.6. Giemsa staining

Three replicates were prepared for each concentration. The samples obtained from each study group were spread on slides and fixed with methanol for 10 min. The slides were then stained with May-Grünwald for 1 min and washed with distilled water. Giemsa (1:1) for 5 min at room temperature, washed with water, and viewed by light microscopy.

### 2.7. Scanning electron microscopy

For the morphological analysis, HL60 cells exposed to NB and NB-mediated PDT with a concentration that corresponded to the effective dose (12.5 μM) were fixed in 3% glutaraldehyde. Subsequently, the cells were transferred to a microscope slide and dehydrated in an ascending solution of ethanol. The samples were coated with palladium-gold and monitored on a JSM 5600 scanning electron microscope.

### 2.8. Mitochondrial membrane potential (ΔΨm) assay

The loss of mitochondrial membrane potential was quantitatively determined by flow cytometry using the JC-1 assay according to the manufacturer’s protocol (BD MitoScreen Mitochondrial Membrane Potential Detection Kit, BD, USA). At least 10,000 events were analyzed via flow cytometry (BD Accuri C6 Plus, USA).

### 2.9. Cell cycle analysis

SubG1 apoptotic peak analysis of all study groups was evaluated using the BD Cycletest Plus DNA kit (BD Biosciences, USA). At least 10,000 events were acquired for analysis. The percentage of cells in the subG1 phase of the cell cycle was determined by BD Accuri C6 Plus software.

### 2.10. Apoptosis detection using annexin V staining

Cells (1 × 10^6^ cells/sample) were stained by annexin V-FITC (BD Biosciences, USA). The quantification of apoptosis induced by NB-mediated PDT in HL60 cells was performed via flow cytometry and software (BD Accuri C6 Plus, USA). The rate of apoptosis was quantified.

### 2.11. Total reactive oxygen species (ROS) assay

Intracellular ROS generation was evaluated using a ROS assay stain fluorescence probe (APB Biosciences, Rockville, USA). It was used to determine some species of intracellular ROS generation in terms of increased fluorescence and those that are highly resistant to autoxidation. H2DCFDA which is a unique fluorescent probe was used to detect reactive oxygen species. Nonfluorescent and cell-permeant H_2_DCFDA is chemically reduced acetylated form of 2′,7′-dichloro-fluorescein (DCF). When H_2_DCFDA makes an entrance into cells, the acetate groups are removed by intracellular esterases to form H_2_DCF. H_2_DCF is held in cells. Oxidation of H_2_DCF by reactive oxygen species yields fluorescent DCF. The cells were preloaded with the probe for 30 min prior to PDT application at 37 °C, and this probe was present during irradiation. Before the PDT application process, the cells were preloaded with probe for 30 min at 37 °C, and this probe was present during irradiation. It was monitored by flow cytometry using excitation sources and filters appropriate for fluorescein (FITC). The fraction of fluorescence-positive cells, as the proportion of cells containing intracellular ROS, was measured according to the Total ROS Assay Kit protocols provided by the manufacturer by flow cytometry (BD Accuri C6 Plus).

### 2.12. Analysis of CD11b expression by flow cytometry

The samples, which were obtained from untreated control groups and treated HL60 cells at low concentrations, were washed with PBS, and PE-conjugated antihuman CD11b antibody (BD Biosciences, USA) was added to the cells and incubated at 25 °C for 45 min followed by washing with PBS. These samples were analyzed on the BD Accuri C6 Plus Flow cytometer. The results were represented using histograms via the BD Accuri C6 Plus software.

### 2.13. Data processing and statistical calculation

All experiments were repeated at least three times in triplicate wells. The results were expressed as the mean ± standard deviation (SD). All data was presented and analyzed using SPSS 17. One-way ANOVA analysis of variance followed by a Tukey post hoc test was used for data analysis. p-values less than 0.05 were considered to be significant.

## 3. Results

### 3.1. NB-mediated PDT reduced the viability and volume of HL60 leukemia cells

**Figure 1:**
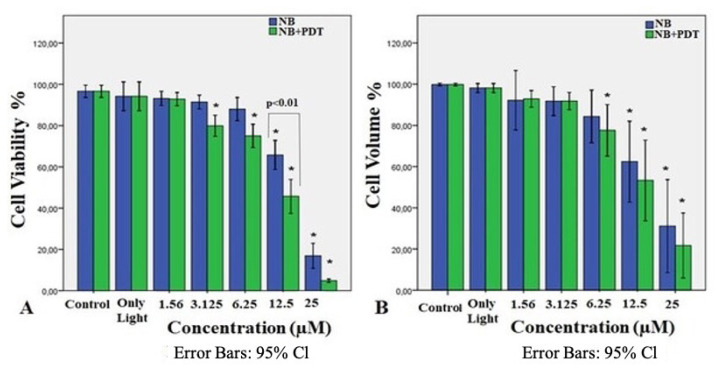
All experiments were repeated at least 3 times. Graphical results are given as mean + SD of three experiments. The PDT effect on cell viability was potentiated by the addition of NB. HL60 cells were treated with NB (25, 12.5, 6.25, 3.25, and 1.56 μM) and NB mediated PDT (0.47 mW/cm^2^, and fluence of 0.84 J/cm^2^ at 30 min) before the percent of cell viability was generated by tyrpan blue exclusion assay (A) and cell volume assay (B). ^*^ indicates statistical significance compared to control group (p < 0.01).

In the present experiment, the dose responses of NB-mediated PDT were investigated in HL60 cells which are exposed to various concentrations of NB (25, 12.5, 6.25, 3.25, and 1.56 μM) for 24 h. The effect of NB-mediated PDT on the viability of the HL60 cells was detected using the trypan blue exclusion test and cell volume assay. As shown in Figure 1, NB-mediated PDT reduced the viability and volume of the HL60 cells in vitro in a dose-dependent manner when compared with untreated cells. These results suggest that NB-mediated PDT was cytotoxic to human leukemia cells, and a concentration of 12.5 μM (IC50 value) was determined as the most appropriate dose, where the low dark toxicity of NB, and also the combination of NB and PDT is toxic (p < 0.01) for further apoptosis-related studies.

### 3.2. NB-mediated PDT induces morphological and ultrastructural alterations in HL60 cells

**Figure 2:**
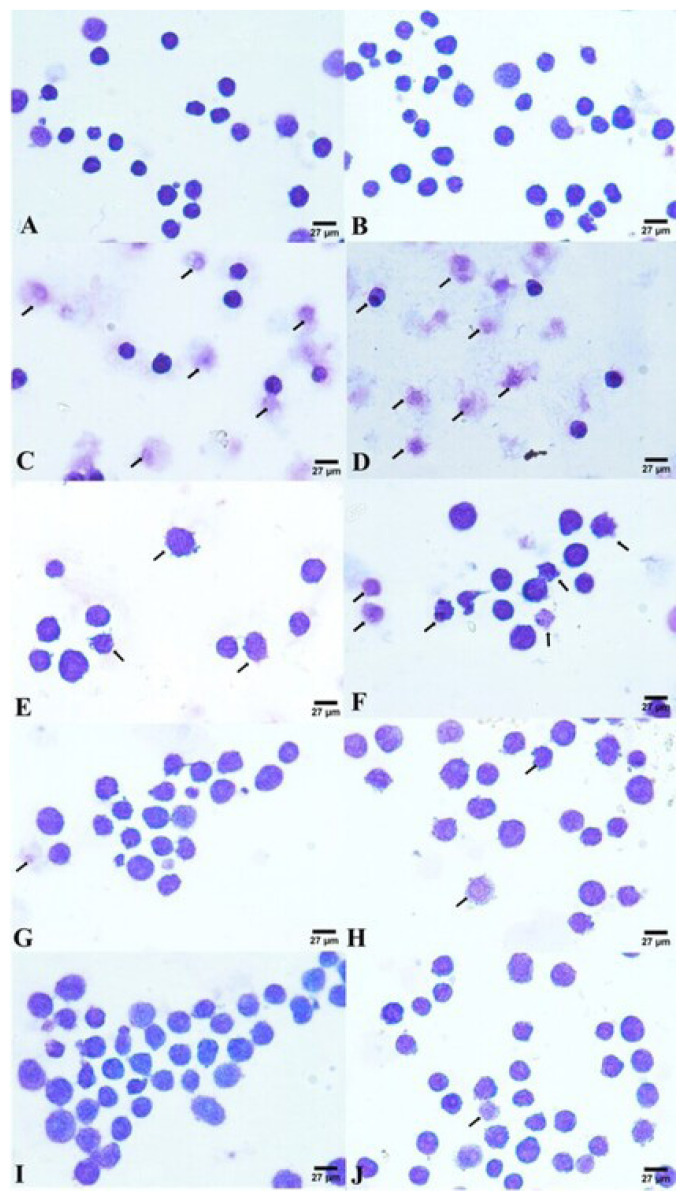
Morphological changes of the cells by Giemsa staining observation for 24 h. No obvious cell damage was observed in the control group. Apoptosis occurred in the treatment group. Arrows indicate affected and damaged cells. Scale bars represent 27 μm; original magnification 100×. Images were analyzed by ImageJ software. Three independent experiments were carried out. (A) Control, untreated HL60 cells; (B) Control, treated with only light; (C) HL60 cells treated with 25 μM NB; (D) HL60 cells treated with light combined with 25 μM NB; (E) HL60 cells treated with 12.5 μM NB; (F) HL60 cells treated with light combined with 12.5 μM NB; (G) HL60 cells treated with 6.25 μM NB; (H) HL60 cells treated with light combined with 6.25 μM NB; (I) HL60 cells treated with 3.125 μM NB; and (J) HL60 cells treated with light combined with 3.125 μM NB.

**Figure 3:**
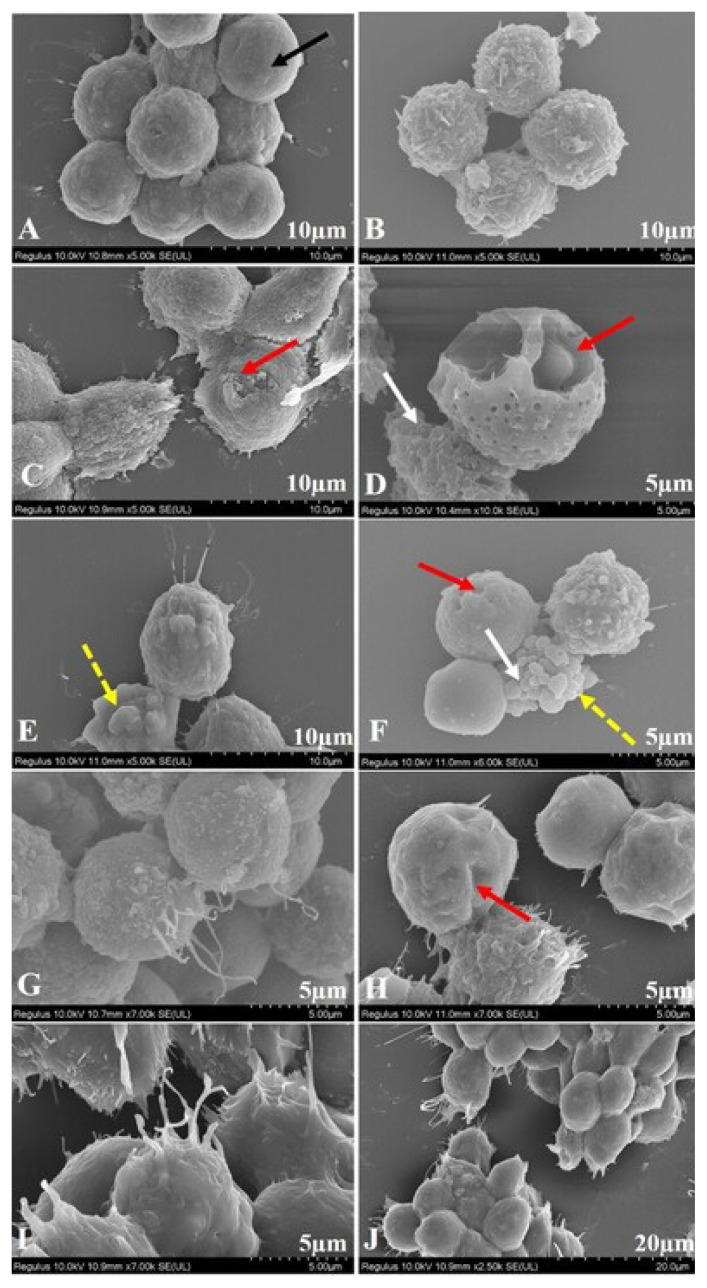
Ultrastructural alterations of the cells by SEM observation for 24 h. No obvious cell damage was observed in the control group. Apoptosis occurred in the treatment group. Black solid arrow indicates live cell, red solid arrow indicates apoptotic cell with holes, yellow dashed arrow indicates shrinking cell with apoptotic bodies and white solid arrow indicates broken cell membranes. (a) Control, untreated HL60 cells; (b) Control, treated with only light; (c) HL60 cells treated with 25 μM NB; (d) HL60 cells treated with light combined with 25 μM NB; (e) HL60 cells treated with 12.5 μM NB; (f) HL60 cells treated with light combined with 12.5 μM NB; (g) HL60 cells treated with 6.25 μM NB; (h) HL60 cells treated with light combined with 6.25 μM NB; (i) HL60 cells treated with 3.125 μM NB; and (j) HL60 cells treated with light combined with 3.125 μM NB. Scale bars of D, F, G, H, I micrographs represent 5 μm; Scale bars of A, B, C, E micrographs represent 10 μm; Scale bar of J micrograph represents 20 μm. Three independent experiments were carried out.

Giemsa staining and scanning electron microscopy (SEM) were employed to assess morphological alterations in the HL60 cells induced by NB-mediated PDT. Apoptotic cells in treatment group were detected by Giemsa staining (Figure 2). SEM analysis of the control cells revealed normal cell structure without cell damage. However, in the treatment group, some cells had shrunk in volume and showed typical apoptotic properties such as apoptotic bodies, holes, and broken cell membranes in a concentration-dependent manner. A comparative morphological analysis of the SEM data is shown in Figure 3. It also shows that hole induction was associated with membrane blebbing. These morphological changes are considered to be signs of leukemia cell apoptosis (Ghoneum and Gimzewski, 2014).

### 3.3. NB-mediated PDT induces annexin V-positive apoptosis in HL60 cells

**Figure 4:**
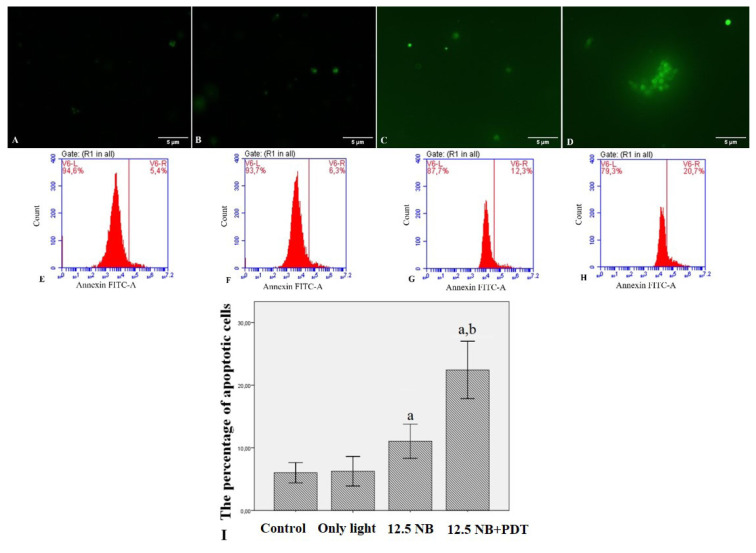
The apoptosis was determined by flow cytometry and fluorescent microscopy. A significant increase in annexin V-binding cells with green fluorescence was detected in treated cells compared to control untreated cells. HL60 cells were treated with NB (12.5 μM), light, and NB-mediated PDT. Fluorescent microscopy images in FITC filter. Scale bars represent 5 μm; Original magnification 20×. Images were analyzed by ImageJ software. Three independent experiments were carried out. ((A) Control, untreated HL60 cells; (B) Control, treated with only light; (C) Dim annexin V binding HL60 cells treated with 12.5 μM NB; (D) Bright annexin V binding HL60 cells treated with light combined with 12.5 μM NB)); Flow cytometry histograms for 24 h ((E) Control, untreated HL60 cells; (F) Control, treated with only light; (G) HL60 cells treated with 12.5 μM NB; (H) HL60 cells treated with light combined with 12.5 μM NB)); (I) Graphical presentation of the percentage of the annexin V positive apoptotic cell. ^a^ indicates statistical significance compared to control group; ^b^ indicates statistical significance between NB and light combined with 12.5 μM NB group.

Several studies have demonstrated that photosensitizer-mediated PDT can induce apoptosis in various cancer types. Therefore, the role of apoptosis in enhancing the antileukemic effect of PDT was sought to be determined by NB. Apoptotic changes were detected by flow cytometry using AnnexinV-FITC staining (Figure 4). HL60 cells were treated with NB (12.5 μM), light, or their combination for 24 h. As shown in Figure 4, NB increased the percent of apoptotic cells compared to untreated control cells and from 6.0 ± 0.7% to 11.0 ± 1.1% in the HL60 cell line, while only light treatment did not increase the apoptosis from 6.0 ± 0.7% to 6.2 ± 0.9%. The combination of NB and PDT treatment produced significantly more pronounced apoptosis compared with other study groups (22.4 ± 1.8% with p < 0.05).

### 3.4. NB-mediated PDT induces subG1 apoptotic peak in HL60 cells

**Figure 5:**
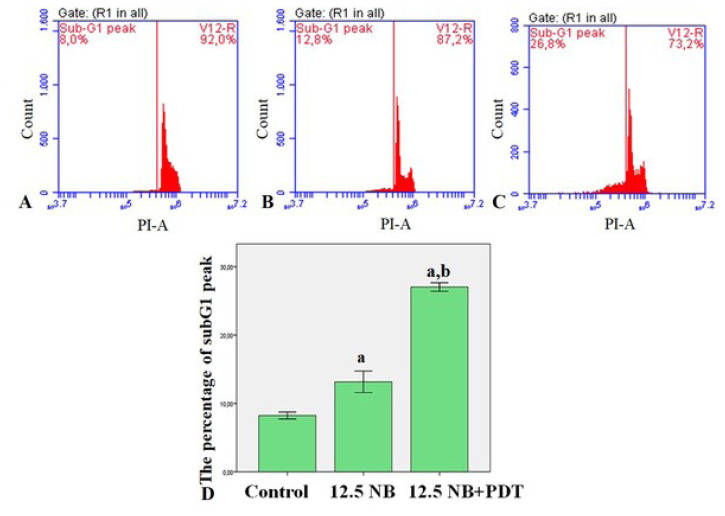
SubG1 apoptotic peak was determined by flow cytometry. HL60 cells were treated with NB (12.5 μM) and NB-mediated PDT. Flow cytometry histograms for 24 h ((A) Control, untreated HL60 cells; (B) HL60 cells treated with 12.5 μM NB; (C) HL60 cells treated with light combined with 12.5 μM NB)); (D) Graphical presentation of the percentage of the SubG1 peak apoptotic cell. ^a^ indicates statistical significance compared to control group; ^b^ indicates statistical significance between NB and light combined with 12.5 μM NB group.

The formation of apoptosis was also confirmed by recording the cell cycle histogram. In the cell cycle histogram of HL60 leukemia cells, the presence of characteristic peak at subG1 is due to cell death such as apoptosis or necrosis. Flow cytometry was used to record the apoptotic behavior of HL60 leukemia cells during NB-mediated PDT at a concentration of 12.5 M for 24 h as shown in Figures 5A and 5B. The percent change in the subG1 apoptotic peak of HL60 cells was calculated as 8.2±0.2%, 13.2± 0.6%, and 27.0± 0.3% for the untreated control group, 12.5 M NB, and light combined with 12.5 M NB, respectively.

### 3.5. NB-mediated PDT induces leukemia cell mitochondrial membrane depolarization

**Figure 6:**
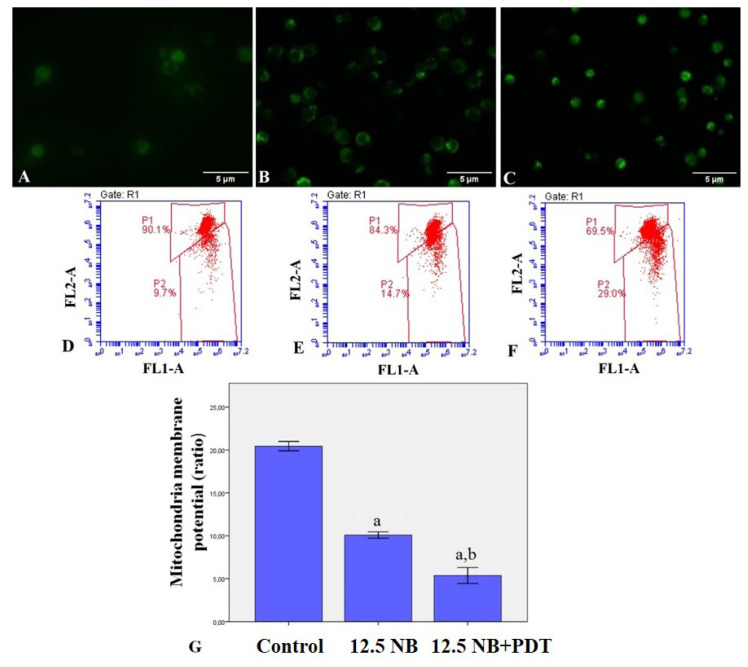
Effect of only NB and NB and light combination on the mitochondria-mediated apoptosis of promyelocytic leukemia cells. JC-1 dye can be used as a measure of mitochondrial membrane potential. Mitochondrial depolarization is indicated by a reduction in the red to green fluorescence intensity ratio. FL1-A shows the FITC green channel and FL2-A shows the red channel. The aggregate green monomeric form was observed in green filter under a fluorescence microscope at 40× magnification. Scale bars represent 5 μm; original magnification 40×. Images were analyzed by ImageJ software. ((A) Control, untreated HL60 cells; (B) HL60 cells treated with 12.5 μM NB; (C) HL60 cells treated with light combined with 12.5 μM NB; Flow cytometry histograms for 24 h ((D) Control, untreated HL60 cells; (E) HL60 cells treated with 12.5 μM NB; (F) HL60 cells treated with light combined with 12.5 μM NB)); (G) Graphical presentation of the ratio of mitochondria membrane potential changing in HL60 cells. ^a^ indicates statistical significance compared to control group; ^b^ indicates statistical significance between NB and light combined with 12.5 μM NB group (p < 0.01).

To further characterize the apoptosis induced by NB-mediated PDT in promyelocytic leukemia cells, whether it caused mitochondrial apoptotic changes in HL60 cells was investigated by flow cytometry and fluorescence microscopy. JC1 staining showed that treatment with NB and light combination for 24 h induced mitochondrial membrane depolarization of these cells (Figure 6). However, induction of mitochondrial membrane depolarization by NB and light combination treatment was observed more clearly than only NB treatment in HL60 cells. Figure 6 depicts that the ratio of ΔΨm changing in untreated control cells, only NB-treated cells, and combination (NB+PDT)-treated cells was 20.5 ± 0.2, 10.0 ± 0.2, and 5.4 ± 0.4, respectively.

### 3.6. NB-mediated PDT induces ROS generation in HL60 cells

**Figure 7:**
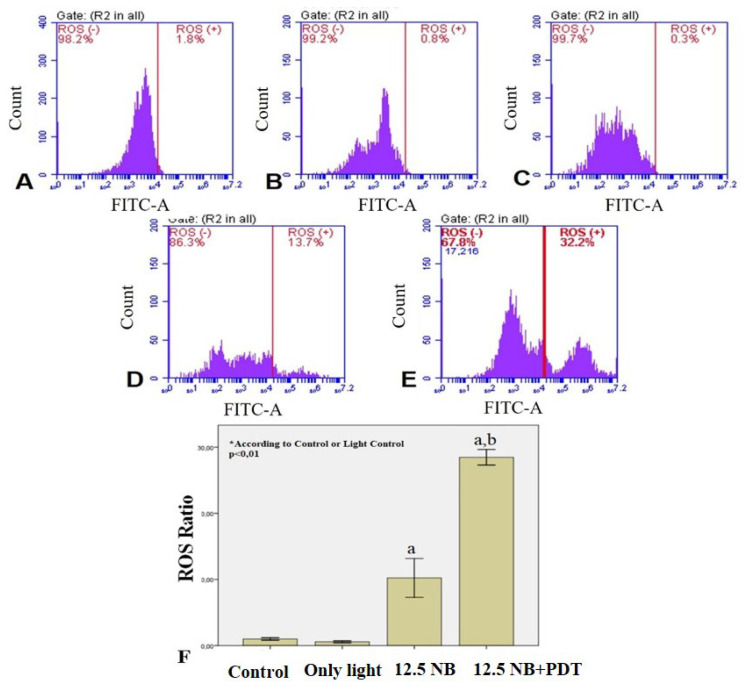
All experiments were repeated at least three times. Graphical results are given as mean + SD of three experiments. Effect of NB mediated PDT on excessive ROS generation in HL60 cells. ROS was measured by staining the cells with H_2_DCFDA cellular ROS detection assay kit according to the manufacturer’s instructions. ROS generation was measured by flow cytometer (A) Unstained cells (B) Control, untreated HL60 cells; (C) Control, treated with only light; (D) HL60 cells treated with 12.5 μM NB; (E) HL60 cells treated with light combined with 12.5 μM NB; (F) Graphical presentation of the ratio of ROS generation in HL60 cells. ^a^ indicates statistical significance compared to control group; ^b^ indicates statistically significance between NB and light combined with 12.5 μM NB group (p < 0.01).

NB-mediated PDT remarkably elevated the generation of ROS in HL60 cells compared to that in control cells, but only NB treatment did not affect that well (Figure 7). These results imply that the generation of ROS is involved in the regulation of apoptosis caused by the PDT with NB combination in myeloid leukemia cells.

### 3.7. NB-mediated PDT had no effect on HL60 cell differentiation by CD11b staining

**Figure 8:**
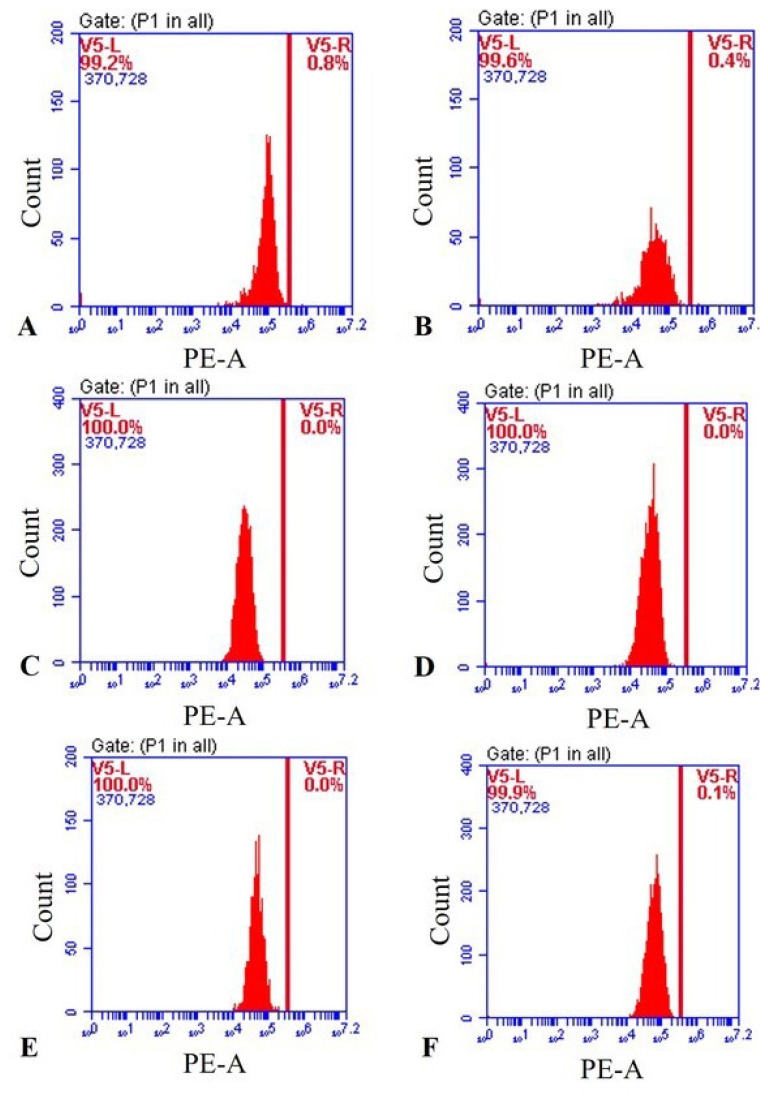
Effect of only NB, NB, and light combination on the HL60 leukemia cell differentiation. Flow cytometry histograms for 96 h [(A) Control, untreated HL60 cells; (B) only light control; (C) HL60 cells treated with 1.56 μM NB; (D) HL60 cells treated with light combined with 1.56 μM NB); (E) HL60 cells treated with 3.125 μM NB; (F) HL60 cells treated with light combined with 3.125 μM NB)].

Granulocytic lineage as characterized by CD11b expression, a marker of differentiation, was detected. Taken together, these results indicate that differentiation-inducing activity was not determined by NB-mediated PDT and may be assessed with other differentiation therapeutics. No change in the presence of CD11b positive cells could be detected in the only NB treated and also NB combined with light treated HL60 cells from day 5 by measuring the mean fluorescent intensity and the percentage, when compared to the untreated control (Figure 8A and 8B).

## 4. Discussion

There are some problems in the application of the treatments routinely used in leukemia therapy. The side effects of high doses of radiotherapy against our normal cells are prominent (Shimada et al., 2014). Chemotherapy may have similar side effects and the possibility of drug resistance (Lv et al., 2019). Hematopoietic stem cell transplants are one of the most frequently used methods in the treatment of leukemia. For this therapy, there are significant risks in terms of recipient and donor (Lawitschka and Peters, 2018). Not all therapeutic approaches are effective in the complete destruction of leukemia cells. Photodynamic therapy is a minimally invasive and complementary therapeutic procedure for cancers and other diseases (Kennedy et al., 1996). Skin tumors, head and neck tumors, digestive system tumors, intraperitoneal malignancies, urinary system tumors, and small cell tumors (Dolmans et al., 2003). Clinical studies reveal that PDT can prolong survival and significantly improve quality of life in patients with inoperable cancer (Wang et al., 2021). Several previous studies have shown that PDT can be applied to leukemia cells through different photosensitizers (Zhang et al., 2002; Xu et al., 2015; Sando et al., 2021). This application has a cytotoxic effect on leukemia cells and also enables them to differentiate into phagocytic cells (Smetana et al., 2000). The effectiveness of photodynamic therapy varies depending on the photosensitizer applied, the light source, and the type of treatment (Lilge et al., 1998). The severity of the phototoxic effect is directly proportional to the dose of the drug and the radiation. It has been reported that the dose of the administered drug should be reduced as much as possible in order to avoid side effects, especially due to the ideal agent to be used in clinical applications, but photosensitizers with high efficacy but low skin toxicity, which do not require longer-term radiation therapy to achieve the same effect, should be used (Wormald et al., 2003). Recently, ex vivo applications such as immunotherapy and replacement therapy have been used in leukemia patients. PDT application has fewer side effects and is a noninvasive method. Recent research has demonstrated that this treatment is effective in a variety of leukemia cell lines (Yuan et al., 2019). This treatment could be used to remove leukemic cells from bone marrow intended for autologous transplant.

To demonstrate the photodynamic effect on leukemia cells, NB-mediated PDT experiments were performed using the HL60 cell line in this study. The results showed that both NB and light applications alone did not cause a significant cytotoxic effect on this cell line. NB combined with PDT, affected these cell lines in a concentration-dependent manner. After incubation with 12.5 μM NB and light treatment with 0.84 J/cm^2^, the light enhanced the approximately 50% inhibition of the leukemia cells. The HL60 cell line is widely used in chronic myeloid leukemia studies. To our knowledge, this cell line is used for the first time in the research of NB-mediated PDT. Philchenkov et al. demonstrated that ALA and fotolon^®^-mediated PDT resulted in dose-dependent cell death in human T cell acute lymphoblastic leukemia lines (Philchenkov et al., 2014). Annexin-V staining was used to investigate the role of photodynamic therapy in cell apoptosis. Annexin V staining, which binds to phosphatidylserine passing from the cytoplasmic surface to the outer surface of the plasma membrane, which is one of the indicators of apoptosis, is an important method for detecting early stage apoptosis. The results of PI staining with annexin V could not be evaluated, since NB, which was used as a photosensitizer in the study, overlapped at a wavelength similar to the PI fluorescent dye. Thus, only annexin V was used to identify apoptotic cells. In this study, most of the apoptotic cells observed in HL60 cells were stained with annexin V, suggesting that NB-mediated PDT treatment is effective in the action of the membrane phospholipid phosphatidylserine in these cells. Cisáriková et al. reported that Acridin-3,6-dialkyldithiourea hydrochlorides (AcrDTUs)-mediated PDT increased cytotoxicity and arrested the cell cycle in the subG0 phase on mouse leukemia line L1210 (Cisáriková et al., 2016). In another study, Feuser et al. suggested that PDT can be an excellent alternative for the treatment of leukemia (Feuser et al., 2016). Grebenova et al. demonstrated that PDT inhibits proliferation and viability and causes arrest in the interphase of the cycle of human promyelocytic leukemia HL60 cells and human erythroleukemia HEL cells (Grebenová et al., 1997).

However, the change in mitochondrial membrane potential with JC-1 staining was investigated by flow cytometry. It was determined that the mitochondrial membrane was affected in the NB+PDT applied cells compared to the only light and only NB applied groups. These findings demonstrated that NB and light combination therapy caused depolarization of the mitochondrial membrane, reducing the viability of the cell and leading to apoptosis. There is an important relationship between mitochondria and ROS production. To detect intracellular ROS generation after NB-mediated PDT, and H_2_DCFDA probe was used and all study groups were monitored by flow cytometry. The results showed that cells treated with NB+PDT had higher levels of ROS than cells treated with NB alone. In this study, ROS is sharply produced as a result of NB+PDT application, showing that cytoplasmic damage is induced. NB-mediated PDT could produce strong damage directly to the cytoplasm during the phototherapy process. Other studies demonstrated that PDT can trigger apoptosis, reactive oxygen species formation, and loss of mitochondrial transmembrane potential in HL60 cells in vitro (Ettorre et al., 2010; Moosavi et al., 2016; Salmerón et al., 2018).

The morphological and ultrastructural examinations were investigated via Giemsa staining and SEM methods, respectively. A careful examination of the images, clearly shows that NB and light combination therapy has slightly typical apoptotic cell features, including bubble-like protrusions on the HL60 cell surface and the formation of apoptotic bodies, due to its higher apoptotic potential as compared to only NB, only light treatment, and the untreated control group.

Leukemia is characterized by blocking differentiation at the developmental stage. Furthermore, when any therapeutic approach induces leukemia cell differentiation, they express new genes and molecules undergo morphological changes. Such changes have been proposed as indicators of successful differentiation and can be monitored by microscopic, flow cytometric, and molecular techniques. Morphological changes during differentiation include reduction in cell size, decreased nuclear cytoplasmic ratio, increased nuclear pycnosis and segmentation, decreased cytoplasmic basophilia, replacement of coarse azurophilic granules with smaller specific granules, and changes in gene and protein expression in granulocytic lineages. Undifferentiated HL-60 cells have a generally rounded morphology when viewed by scanning electron microscopy, with pseudopodia that are short to medium length and occasional folds; they show clear changes in morphology (increasing folds and flattening) after differentiation (Fleck et al., 2005). CD11b monoclonal antibody was used in our study for detecting leukemia cell differentiation via flow cytometric methodology. As a result of CD11b staining, no effect of NB-mediated PDT on the differentiation of leukemia cells was detected at the nontoxic dose for 96 h. The differentiation effect and killing efficiency of NB-mediated PDT can be further enhanced by combining treatments with other agents. In addition, the effectiveness of this treatment can be increased by changing the applied light source and dose. Taken together, these results indicate that NB-mediated PDT does not possess differentiation-inducing activity and therefore can be evaluated in differentiation therapy for leukemia in combination with other inducers of differentiation.

This study showed that NB alone, combined with photodynamic therapy, can eradicate chronic myeloid leukemia cell lines, although not very effectively. Future work will focus on investigating the selectivity of NB over leukemia cells and normal bone marrow hematopoietic cells, as well as achieving targeted photodynamic therapy of leukemia cells as a promising treatment. A limitation of this study is the investigation of the photodynamic effect of Nile blue on only one leukemia cell line. Another limitation is the inability to compare with healthy bone marrow hematopoietic cells. In addition to these limitations, cell viability could not be determined by classical colorimetric methods due to the wide absorption band of Nile blue. The cytotoxic effect of treatment on HL60 cells was detected by flow cytometry based on cell volume.

Nile blue as a photosensitizer and its combination with PDT were investigated on HL60 leukemia cells successfully. NB alone did not show significant dark toxicity (in the absence of light) at determined dose (12.5μM) and eliminated these cells in a concentration and light-dose–dependent manner. NB-mediated PDT can predominantly trigger the apoptotic response in these cells. NB-mediated PDT can cause ROS accumulation in this cell line. Induction of mitochondrial membrane potential change showed that NB, together with PDT, also affected the mitochondria of leukemia cells. Cell cycle studies confirmed that NB leads to apoptosis of leukemia cells with an increase in subG1 peak level after irradiation. As a result of CD11b staining, no effect of NB-mediated PDT on the differentiation of leukemia cells was detected at the nontoxic low dose after 96 h. However, these findings from cytotoxicity studies showed that NB is a promising photosensitizer in the photodynamic therapy of leukemia cells and may provide new insights for the application of NB in PDT. By penetrating into malignant cells, NB-mediated PDT may be a helpful method in the purification of the bone marrow. Further in vitro, in vivo, and ex vivo experiments are needed to confirm that NB-mediated PDT treatment of leukemia is effective in clinical practice.
